# Quaternization of high molecular weight chitosan for increasing intestinal drug absorption using Caco-2 cells as an in vitro intestinal model

**DOI:** 10.1038/s41598-023-34888-0

**Published:** 2023-05-16

**Authors:** Ratjika Wongwanakul, Sasitorn Aueviriyavit, Tomomi Furihata, Pattarapond Gonil, Warayuth Sajomsang, Rawiwan Maniratanachote, Suree Jianmongkol

**Affiliations:** 1 Department of Pharmacology and Physiology, Faculty of Pharmaceutical Sciences, Chulalongkorn University, 254 Phayathai Road, Bangkok, 10330 Thailand; 2 National Nanotechnology Center, National Science and Technology Development Agency, 111 Thailand Science Park, Pathum Thani, 12120 Thailand; 3 Laboratory of Pharmacology and Toxicology, Graduate School of Pharmaceutical Sciences, Chiba University, Chiba, Japan; 4 Toxicology and Bio Evaluation Service Center, National Science and Technology Development Agency, Pathum Thani, Thailand

**Keywords:** Chemical biology, Drug discovery, Materials science

## Abstract

Potential use of a quaternized chitosan (MW 600 kDa) with 65% of 3-chloro-2-hydroxypropyltrimethylammonium (600-HPTChC_65_) as an absorptive enhancer was investigated in Caco-2 monolayers. 600-HPTChC_65_ (0.005% w/v) quickly reduced transepithelial electrical resistance (TEER) to the maximum level in 40 min with full recovery within 6 h after removal. Its TEER reduction was corresponded to increased FD4 transport across the monolayers and disrupted localization of tight junction proteins ZO-1 and occludin at the cell borders. 600-HPTChC_65_ was densely localized at the membrane surface and intercellular junctions. This chitosan (0.08–0.32% w/v) reduced the efflux ratio of [^3^H]-digoxin by 1.7- 2 folds, suggesting an increased [^3^H]-digoxin transport across the monolayers. Its binding with P-gp on Caco-2 monolayer increased the signal of fluorescence-labeled anti-P-gp (UIC2) reactivity due to conformational change. 600-HPTChC_65_ (0.32% w/v) had no effect on P-gp expression in the Caco-2 monolayers. These results suggest that 600-HPTChC_65_ could enhance drug absorption through tight junction opening and decreased P-gp function. Its interaction with the absorptive barrier mainly resulted in disrupting ZO-1 and occludin organization as well as changing in P-gp conformation.

## Introduction

**Figure 1:**
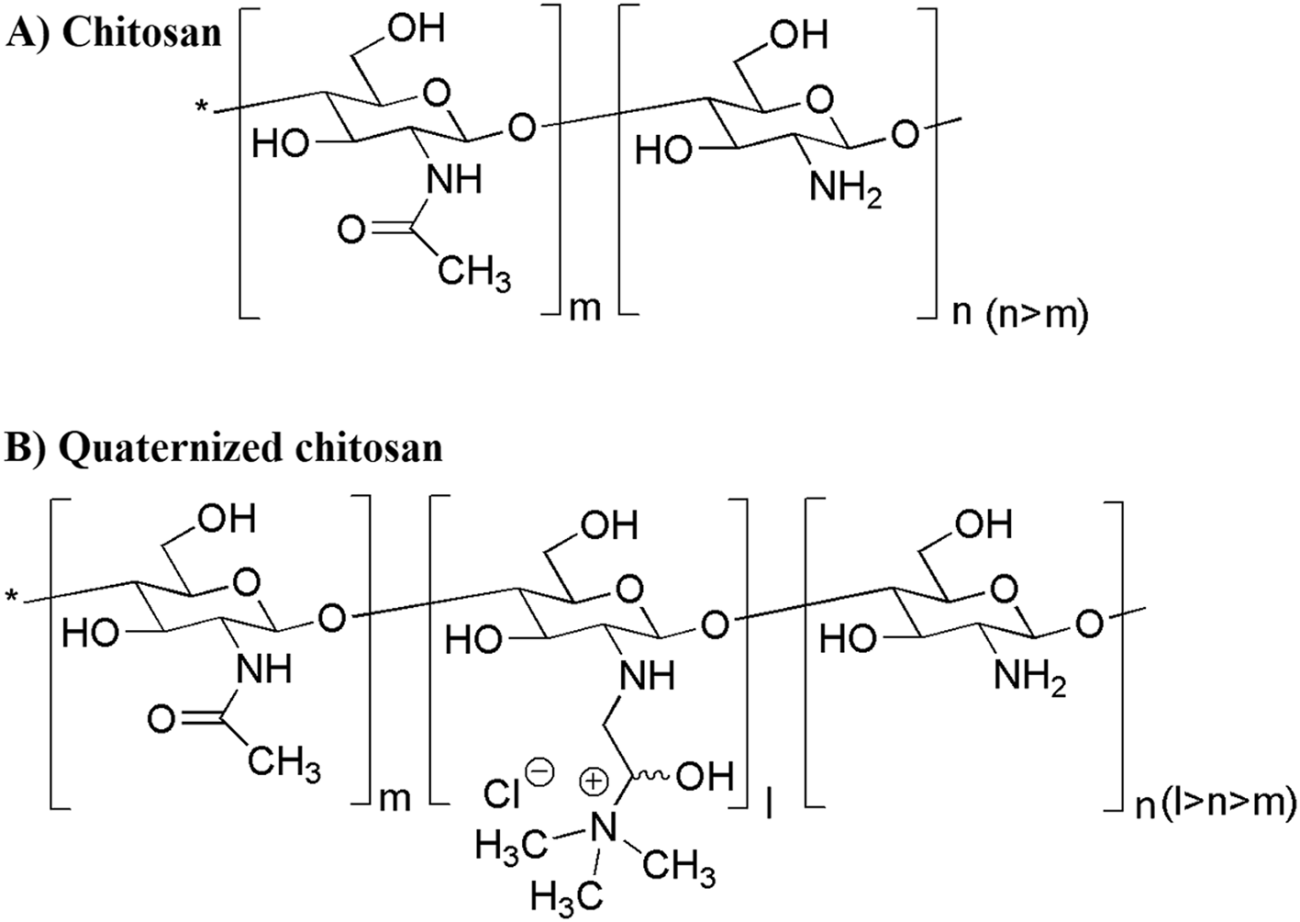
Chemical structures of (**A**) chitosan and (**B**) quaternized chitosan. The percentage of units of chitosan (n), chitin (m) and quaternized (l) are 94%, 6%, 0% for unmodified chitosan, 41%, 6%, 53% for 200-HPTChC_53_ and 29%, 6%, 65% for 600-HPTChC_65_.

The quaternized chitosan derivative (Fig. 1), *N*-(2-hydroxypropyl)-3-trimethylammonium chitosan chloride (HPTChC), was synthesized to improve the solubility and mucoadhesive properties of the chitosan^1,2^. Our previous work demonstrated that 600-HPTChC_65_, generated from 65% degree quaternization (DQ) of chitosan [molecular weight (MW) 600 kDa] with 3-chloro-2-hydroxypropyltrimethylammonium (CTMAC), demonstrated good in vitro biocompatibility with Caco-2 cells, a commonly cell line for in vitro model of small intestine, without alteration of differentiation markers after exposure at 0.005% (w/v) for 4 h/day over 9 days^3^. However, the potential effect of 600-HPTChC_65_ on enhancing oral drug bioavailability needs to be clarified.

Oral drug absorption in the intestine occurs through either paracellular or transcellular transport. Enhancement of the paracellular permeability could improve the absorption of low permeability drugs by loosening the tight junction barrier with drug absorption enhancers, such as cationic polymers (e.g., chitosan and its derivatives)^4^. Previous studies showed that chitosan and 200-HPTChC_53_ (i.e., the HPTChC derived from medium MW chitosan (200 kDa) with a 53% DQ) were able to increase solute transport across intestinal epithelial monolayers via paracellular pathways^5^. Higher MW chitosan was able to increase paracellular permeability and mucoadhesion better than low MW chitosan due to its longer chain structure and higher density of positively charged moieties present on the molecule^6^. In addition to the molecular weight, the degree of substitution with other molecules such as oleic acid in chitosan increased the ferrous ions absorption in Caco-2 cells compared to unmodified chitosan^7^. Modification of chitosan has been widely used as oral drug delivery for improve the stability and efficacy such as ferrous ions and insulin^7–9^. In this regard, it would be of interest to investigate potential effects of the modified high MW chitosan (600-HPTChC_65_) and its related mechanism(s) on the paracellular permeability across the intestinal barrier.

Regulation of a tight junction opening involves the formation of tight junction strands and reorganization of tight junction proteins, such as zonular occludens-1 (ZO-1) and occludin^10^. Several signaling pathways, involving myosin light chain (MLC) phosphorylation and protein kinase C (PKC) also play a role in the physiological tight junction opening^11^. It was reported that inhibition of MLC kinase (MLCK) and PKC in Caco-2 cells prevented the change of tight junction organization^12,13^. Furthermore, the interaction between chitosan and membrane integrin was able to initiate the signaling of a tight junction opening, such as phosphorylation of focal adhesion kinase (FAK) and steroid receptor coactivator (Src)^14^. However, the effect of 600-HPTChC_65_ on tight junction openings and the involvement of intracellular signaling mediated by MLC phosphorylation, PKC and tyrosine kinases are unknown.

A drug efflux pump P-glycoprotein (P-gp) in the intestine has been known to play an important role in the oral bioavailability by limiting transcellular drug absorption. Interference on P-gp function could increase plasma drug level particularly to drugs that are P-gp substrates^15^. In this regard, application of P-gp suppressive agents as an excipient in drug formulation could have a great benefit in enhancing absorption of those P-gp drug substrates. Several synthetic polymers such as polyethylene glycol 400 (PEG 400), pluronic P85 and vitamin E D-α-tocopheryl polyethylene glycol 1000 succinate, that are known as drug carriers in an oral drug delivery system have been demonstrated to be a potential P-gp inhibitor^16,17^. These polymeric non-absorptive materials have advantages of very minimal pharmacological action. It would be of great interest to determine whether 600-HPTChC_65_ is able to enhance transcellular permeability via suppression of P-gp activity.

In this study, we investigated a potential absorptive enhancer of a synthetic 600-HPTChC_65_ polymer using the in vitro Caco-2 cell monolayer model system. The effects of 600-HPTChC_65_ on paracellular and transcellular permeability across the intestinal barriers was determined focusing on mechanisms related to tight junction and P-gp. Modification of the chitosan with HPTChC into 600-HPTChC_65_ would improve the intestinal permeability property of chitosan for a better excipient in the drug delivery system.

## Results

### Effect of 600-HPTChC_65_ on tight junction permeability as compared to that of 200-HPTChC_53_ and unmodified 600 kDa chitosan

**Figure 2:**
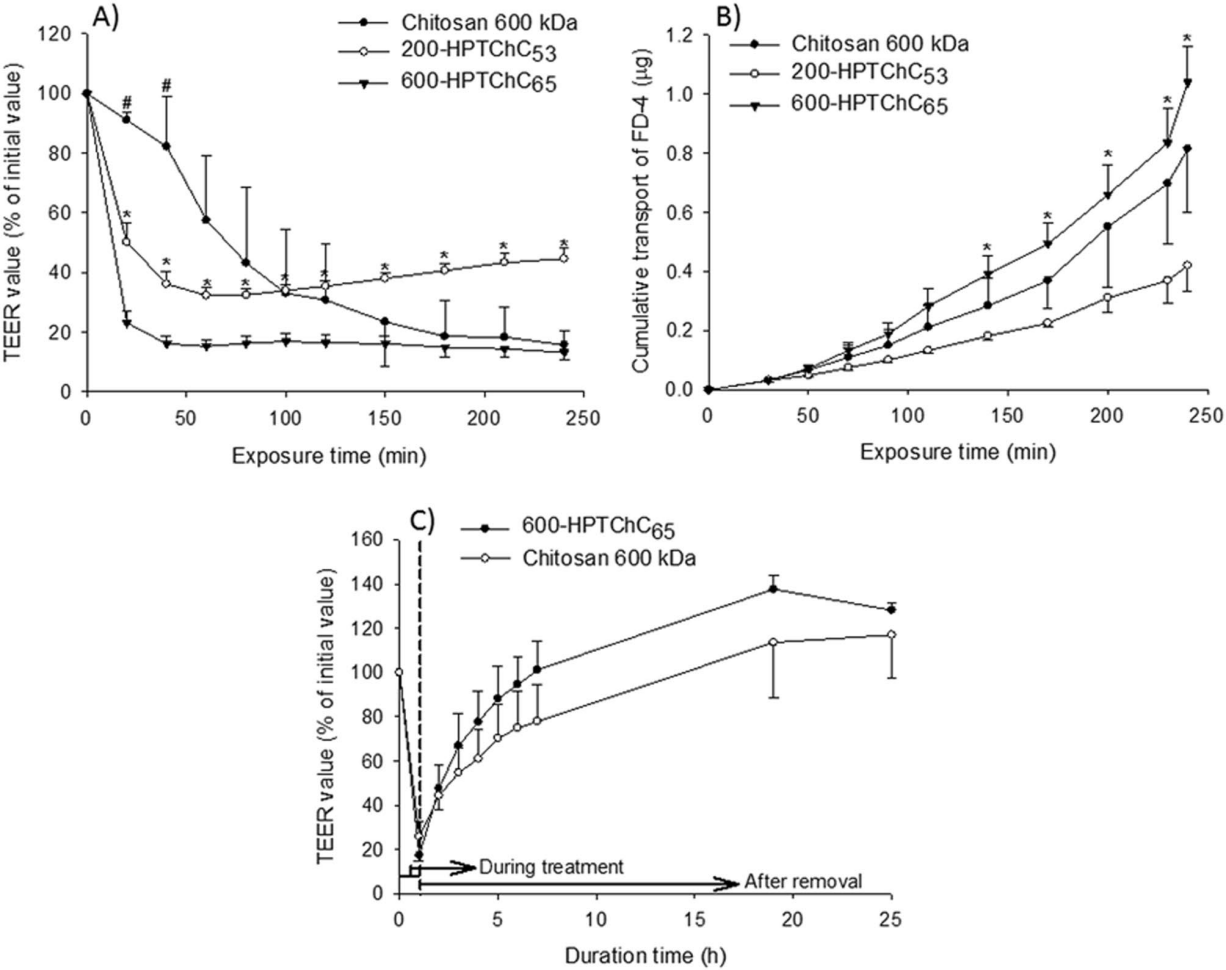
Integrity of tight junction in Caco-2 monolayers after a 4-h exposure to the test chitosan, as shown by (**A**) alteration of the TEER value (% of initial value) and (**B**) amount of FD-4 transport (µg), while (**C**) the return of tight junction integrity following removal of the test chitosan, as shown by increasing TEER values. Data are expressed as the mean ± SEM (n = 4). * and ^#^ represent significant difference (*p* < 0.05) between 600-HPTChC_65_ and 200-HPTChC_53_ or the unmodified chitosan 600 kDa at the same time point, respectively (one-way ANOVA with post-hoc Dunnett’s test).

Chitosan and chitosan derivatives (200-HPTChC_53_ and 600-HPTChC_65_) at the concentration of 0.005% (w/v) were able to markedly decrease the transepithelial electrical resistance (TEER) values of the Caco-2-differentiated monolayers during a 4-h exposure (Fig. 2A). The maximal TEER reduction effect of the two synthetic chitosan derivatives occurred within 40–60 min after exposure, whereas that of the unmodified chitosan 600 kDa reached the maximum after 3-h exposure (Fig. 2A). In addition, the amount of fluorescein isothiocyanate-conjugated dextran (FD-4) transporting across the monolayers gradually increased in the presence of the test compounds, suggesting the loss of tight junction integrity (Fig. 2A and B). At the same concentration of 0.005% (w/v), 600-HPTChC_65_ was more potent than either 200-HPTChC_53_ or the unmodified chitosan 600 kDa in mediating tight junction opening. Increasing the concentration of 600-HPTChC_65_ from 0.005% (w/v) to 0.02% (w/v) did not significantly alter its effect on the tight junction permeability (see Supplementary Fig. S1 online).

After removal of the test chitosan (namely, 600-HPTChC_65_ or the unmodified chitosan 600 kDa), the TEER values gradually returned to a normal level, suggesting that their effects on tight junction opening was reversible (Fig. 2C). Interestingly, the recovery rate of the TEER values of the monolayers treated with 600-HPTChC_65_ was higher than that of the unmodified chitosan 600 kDa.

### Effect of 600-HPTChC_65_ on the localization of tight junction proteins ZO-1 and occludin

**Figure 3:**
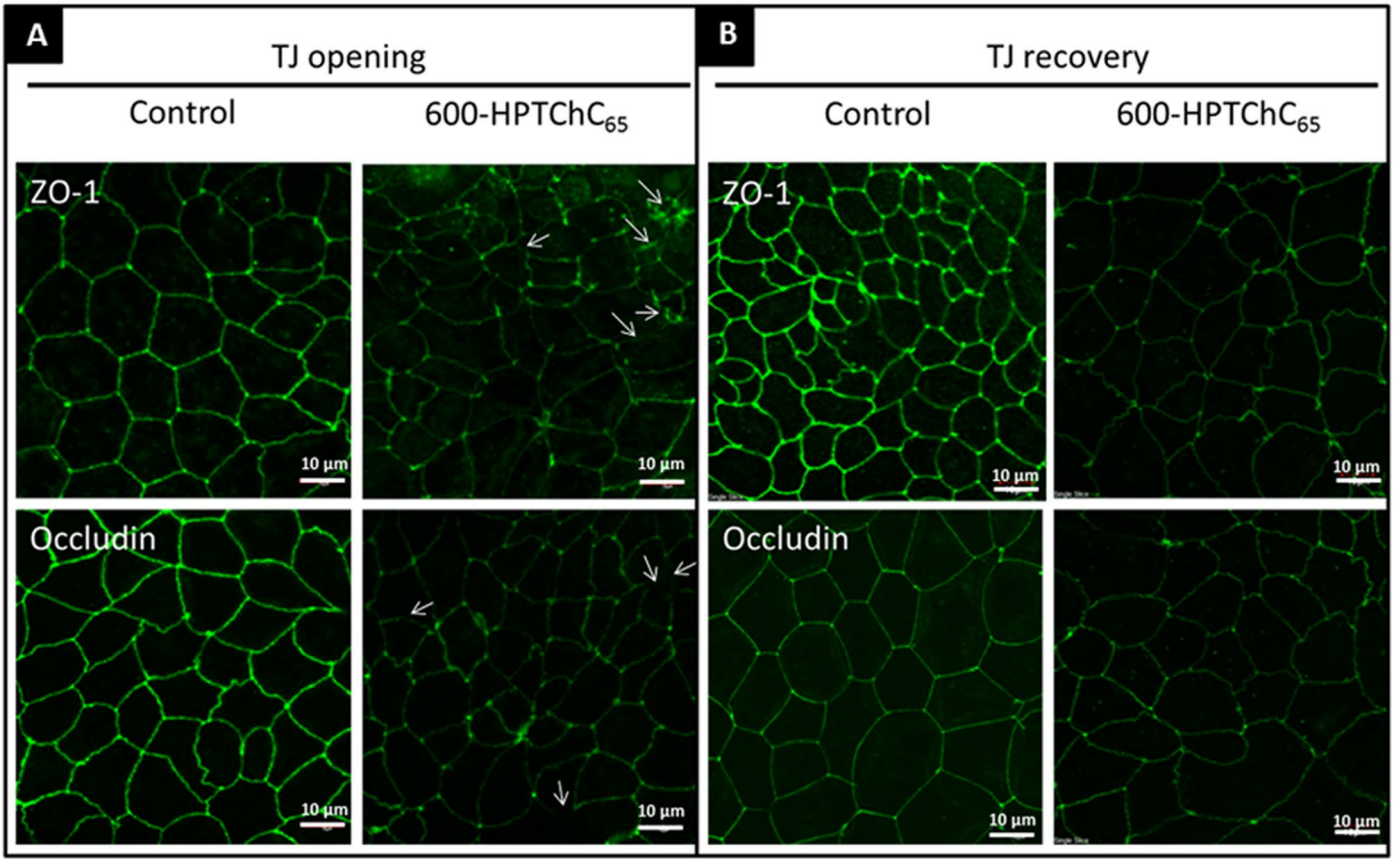
Immunofluorescence staining of tight junction (TJ) proteins, ZO-1 and occludin in Caco-2 monolayers (**A**) after a 3-h exposure to 0.005% (w/v) 600-HPTChC_65_ and (**B**) 24-h after its removal. Arrow shows the disruptions in the expression of ZO-1 and occludin. Images (CLSM; ×40 magnification; scale bar 10 µm) are representative of those seen from at least four fields of view per sample (n = 3).

Immunofluorescent images of the Caco-2-differentiated monolayers demonstrated the continuous distribution pattern of tight junction proteins ZO-1 and occludin at the cell borders (Fig. 3A). Treatment of the monolayers with 0.005% (w/v) 600-HPTChC_65_ for 3 h resulted in a partial disruption of ZO-1 and occludin localization patterns in the intact tight junction structure (Fig. 3A). After removal of the 600-HPTChC_65_ for 24 h, the circumferential expression of ZO-1 and occludin at the cell border was partly restored (Fig. 3B). These results suggested that 600-HPTChC_65_ could induce tight junction opening through interference with the organization of ZO-1 and occludin proteins at cell-to-cell contact sites.

### Tight junction opening by 600-HPTChC_65_ with no involvement of the MLCK, PKC or tyrosine kinase signaling pathways

**Figure 4:**
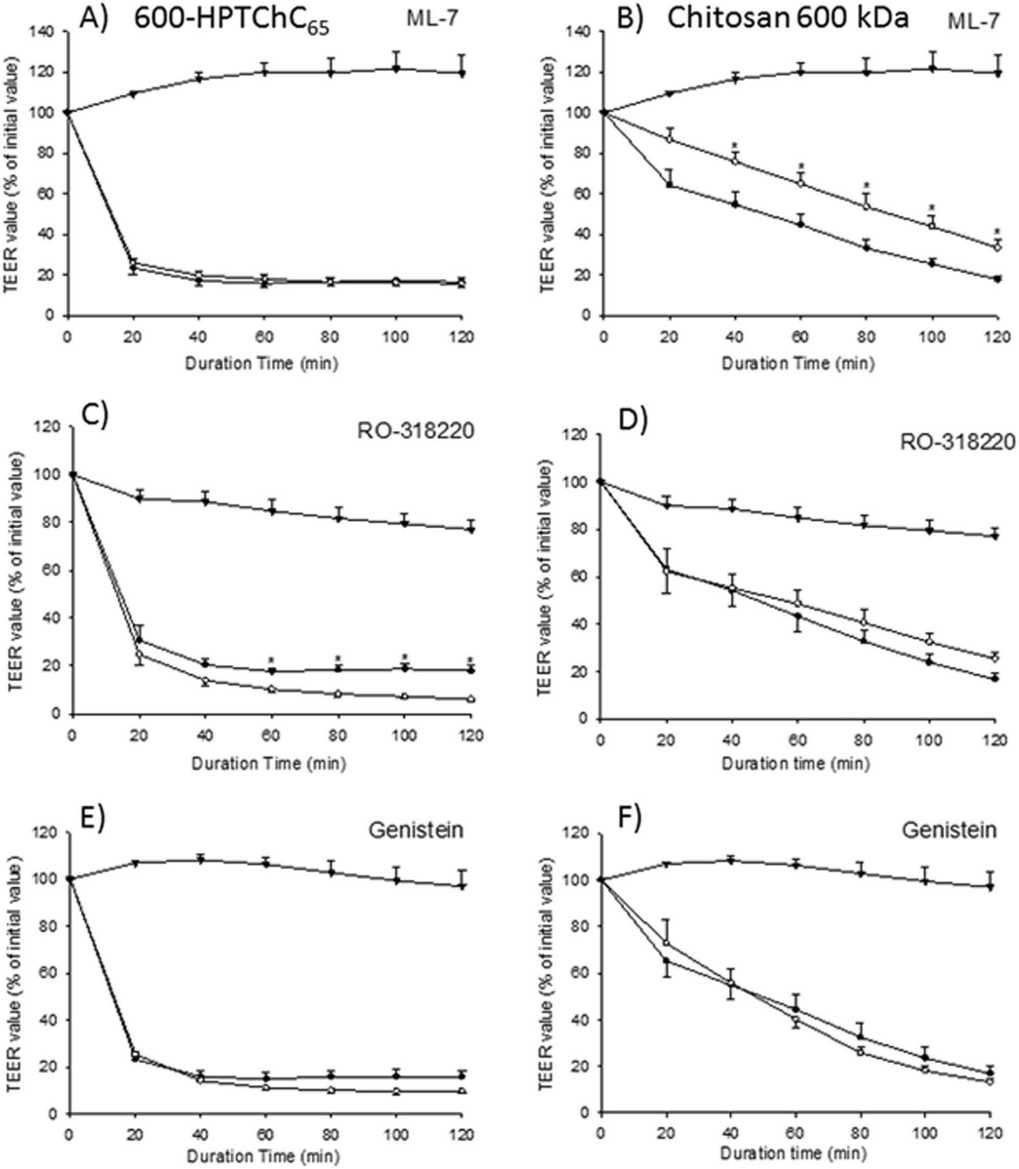
Alteration of the TEER values (% of initial value) after a 2-h treatment with either 0.005% (w/v) (**A, C, E**) 600-HPTChC_65_ or (**B, D, F**) 600 kDa chitosan in the presence of various inhibitors [namely, ML-7 (MLCK inhibitor), RO-318220 (PKC inhibitor), genistein (tyrosine kinase inhibitor)]. Data are expressed as the mean ± SEM (n = 4) Symbols represent: (●) chitosan-treatment, (○) chitosan-treatment in the presence of inhibitor and (▼) inhibitor alone. *p < 0.05 indicates a significant difference between chitosan-treatment groups in the presence (○) and absence (●) of the inhibitor at the same time point (one-way ANOVA with post-hoc Dunnett’s test).

We further investigated the involvement of the MLCK, PKC or tyrosine kinase signaling pathways in tight junction opening by chitosan and 600-HPTChC_65_. As shown in Fig. 4A, C and E, none of ML-7 (a known MLCK inhibitor), RO-318220 (a known PKC inhibitor) or genistein (a known tyrosine kinase inhibitor) prevented the 600-HPTChC_65_-mediated TEER reduction. Inhibition of PKC and tyrosine kinase had no significant effect on the chitosan-mediated tight junction opening (Fig. 4D and F). However, the presence of ML-7 could partially prevent the chitosan-mediated TEER reduction (Fig. 4B). These findings suggested that chitosan could disrupt the integrity of tight junction, in part, via activation of the MLCK pathway. Nevertheless, the mechanism(s) underlying 600-HPTChC_65_-induced tight junction opening did not link to the MLCK, PKC and tyrosine kinase signaling pathways.

### Mucoadhesive property and localization of 600-HPTChC_65_ on Caco-2 monolayers

**Figure 5:**
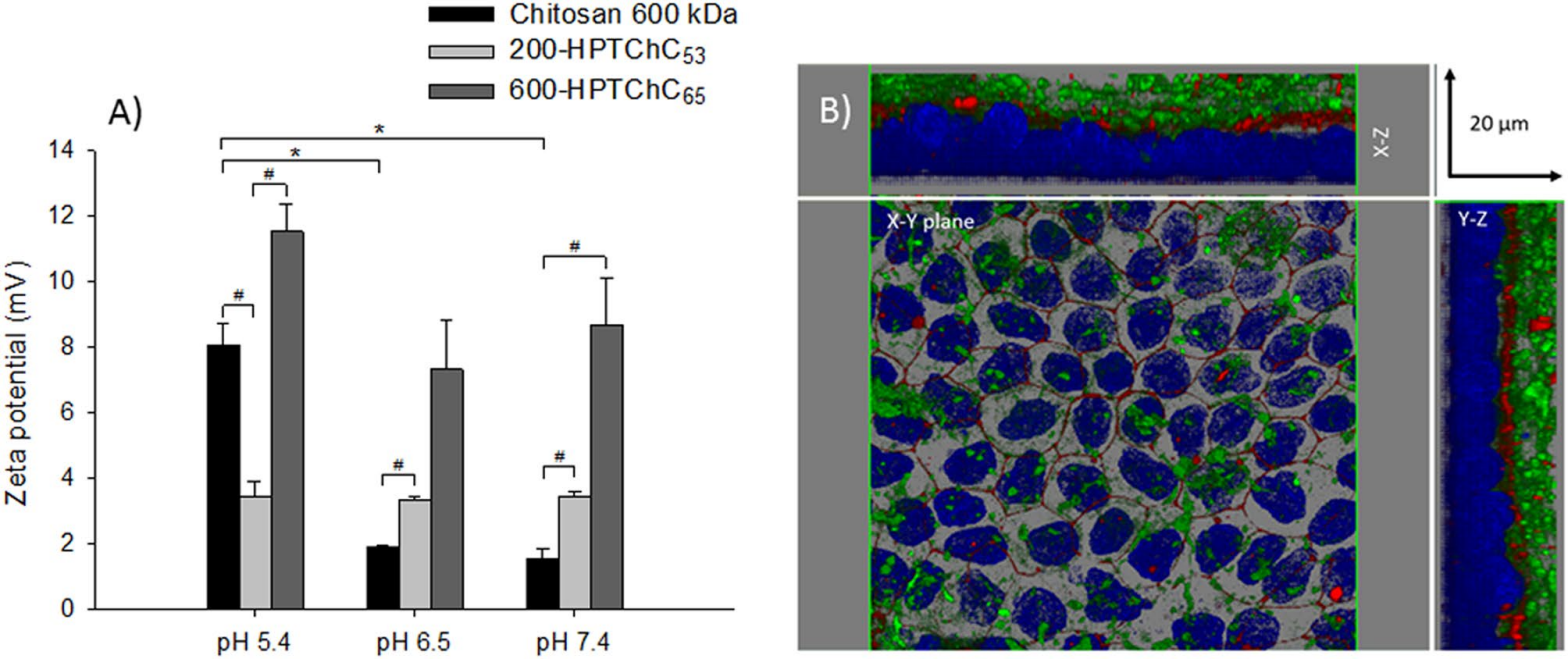
Interaction of 600-HPTChC_65_ with cell membrane and mucin solution. (**A**) Change in the zeta potential of mucin in the presence of the test chitosan at various pH. Data are expressed as the mean ± SEM (n = 3). * and ^#^ represent significant difference (p < 0.05) (one-way ANOVA with post-hoc Dunnett’s test). (**B**) Representative CLSM immunofluorescence images of 600-HPTChC_65_ (0.005% (w/v)) on Caco-2 monolayers after a 3-h exposure of FITC-conjugated 600-HPTChC_65_ (green), ZO-1 (red) and nucleus (dark blue) in cross section (X–Y plane: horizontal plane; X–Z and Y–Z planes: vertical planes) (×40 magnification; scale bar 20 µm).

We further determined the mucoadhesive property of 600-HPTChC_65_, as compared to 200-HPTChC_53_ and the unmodified chitosan 600 kDa, by measuring the alteration of the zeta potential of mucin solution at three different pH values (5.4, 6.5 and 7.4)^18^. As shown in Fig. 5A and Supplementary Table S1 online, the change of the zeta potential of mucin after addition of the unmodified chitosan was greater in the more acidic pH (pH 5.4) than that in pH 6.5 or 7.4. However, the interaction of both chitosan derivatives with mucin was better than that of the unmodified chitosan at pH 6.5 and 7.4, as shown by their higher Δ zeta potential values. The presence of 600-HPTChC_65_ in mucin solution provided the highest change of the zeta potential values at all investigated pH, as compared to 200-HPTChC_53_ and the unmodified chitosan. These findings suggested that 600-HPTChC_65_ produced the highest degree of complex formation with mucin.

Localization of FITC-labeled 600-HPTChC_65_ on Caco-2 monolayers after 3-h exposure was demonstrated with the 3D immunofluorescent images visualized by confocal laser scanning microscopy (CLSM) (Fig. 5B). The FITC-labeled 600-HPTChC_65_ densely localized at the membrane surface and intercellular ZO-1 junctions. However, very small amount of FITC-labeled 600-HPTChC_65_ was observed in an intracellular compartment, suggesting that 600-HPTChC_65_ was scarcely internalized into the cells.

### Inhibitory effect of 600-HPTChC_65_ on P-gp function

Effect of 600-HPTChC_65_ on drug permeability and P-gp function was evaluated by transport of a known P-gp substrate [^3^H]-digoxin across the Caco-2 monolayers. As shown in Table 1, permeability of [^3^H]-digoxin was higher in the basal-to-apical (BL-to-AP) direction than in the apical-to-basal (AP-to-BL) direction, with the efflux ratio (ER) value of 10.1. Verapamil 100 µM interfered with transport of [^3^H]-digoxin in both directions, resulting in the 6.6-fold reduction of the ER value. In the presence of 600-HPTChC_65_ (0.08–0.32% w/v), the permeabilities of [^3^H]-digoxin increased in the AP-to-BL direction and decreased in the BL-to-AP direction (Table 1). Consequently, the ER values of [^3^H]-digoxin were reduced by approximately 1.7–2 folds. These results suggested that 600-HPTChC_65_ might interfere with P-gp function, leading to an increase of [^3^H]-digoxin transport across the intestinal epithelial model.

**Table 1:** Effect of 600-HPTChC_65_ on permeability of [^3^H]-digoxin across the Caco-2 monolayers.

Treatment	P_app(AP-to-BL)_ (× 10^−3^ cm.min^−1^)	P_app(BL-to-AP)_ (× 10^−3^ cm.min^−1^)	Efflux ratio (ER)
Non-treated	0.14 ± 0.01	1.41 ± 0.06	10.1 ± 0.4
Verapamil 100 µM	0.34 ± 0.03^#^	0.52 ± 0.06^#^	1.6 ± 0.1^#^
600-HPTChC_65_ 0.08% (w/v)	0.24 ± 0.02*	1.20 ± 0.05*	5.2 ± 0.6*
600-HPTChC_65_ 0.16% (w/v)	0.21 ± 0.02*	1.14 ± 0.03*	5.6 ± 0.7*
600-HPTChC_65_ 0.32% (w/v)	0.19 ± 0.03	1.08 ± 0.06*	5.8 ± 0.5*

Data are expressed as the apparent permeability coefficient (Papp) and efflux ratio (ER) (mean ± SEM, n = 4). The efflux ratio (ER) is the ratio of P_app(BL-to-AP)_ to P_app(AP-to-BL)_.

* and ^#^ represent significant difference (*p* < 0.05), (Student’s *t*-test for verapamil vs non-treated; one-way ANOVA with post-hoc Dunnett’s test for 600-HPTChC65 vs non-treated).

### Effect of 600-HPTChC_65_ on P-gp ATPase activity

**Figure 6:**
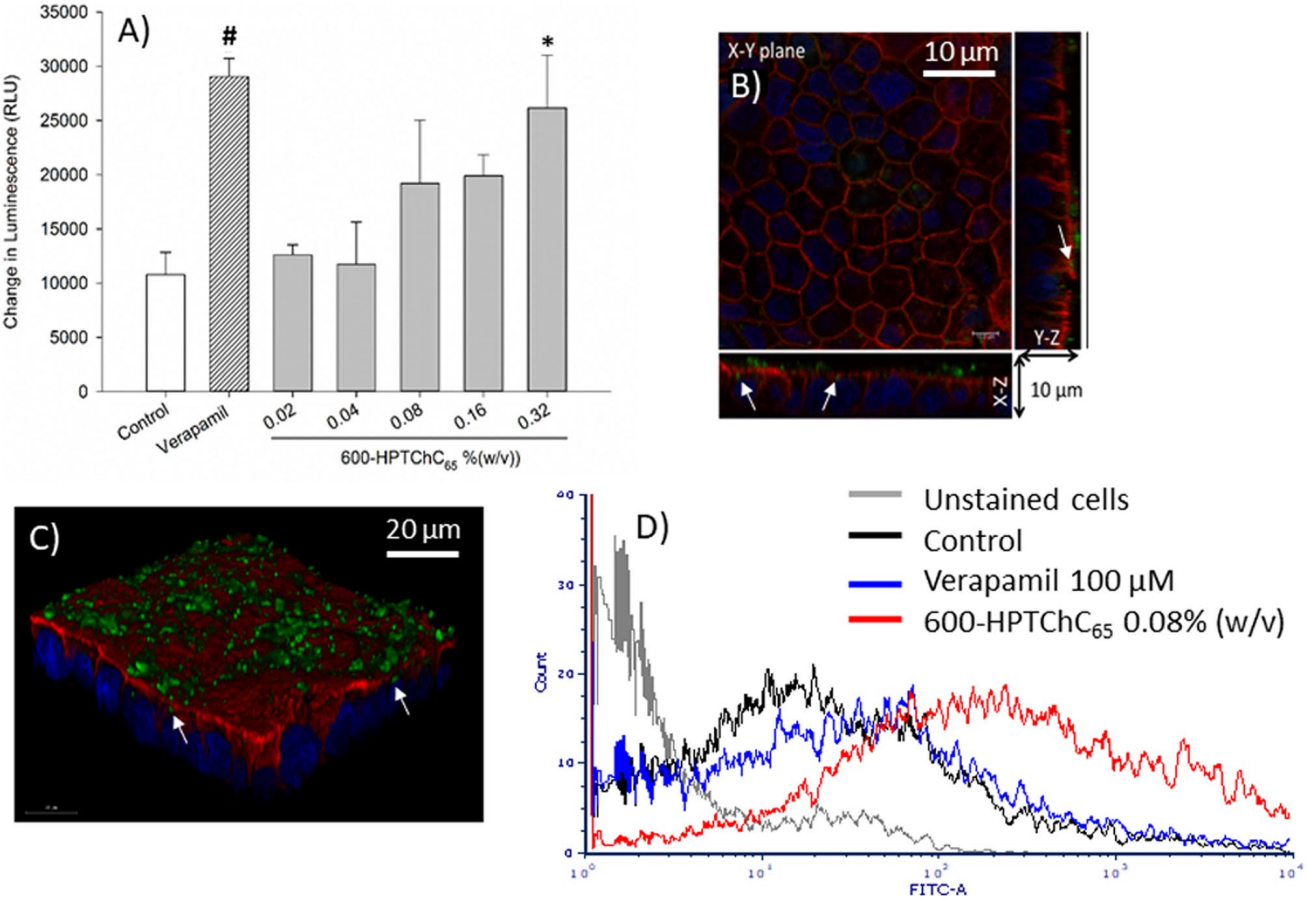
Effects of 600-HPTChC_65_ on ATPase activity and the structure of P-gp. (**A**) ATPase activity in recombinant human P-gp membranes. Data are expressed as the mean ± SEM (n = 3). * and ^#^ represent significant difference (*p* < 0.05), (Student’s *t*-test for verapamil vs control; one-way ANOVA with post-hoc Dunnett’s test for 600-HPTChC_65_ vs control). (**B, C**) Representative CLSM Immunofluorescent images of Caco-2 cells in the presence FITC-labeled 600-HPTChC_65_ (green), Alexa Flour 568 labeled P-gp (red) and nucleus (dark blue). White arrow indicates the overlapping of P-gp and 600-HPTChC_65_ (**B**) 2D and cross-sectional image (scale bar 10 µm) and (**C**) 3D images (scale bar 20 µm) (X–Y plane: the horizontal plane; X–Z and Y–Z planes: the vertical planes). (**D**) Flow cytometry analysis of the binding of FITC-monoclonal antibody UIC2 to P-gp epitope in Caco-2 cells. The cells were incubated with FITC-UIC2 antibody in the absence (control) or presence of either verapamil or 600-HPTChC_65_. The cells without FITC-UIC2 labeling were unstained cells.

Effect of 600-HPTChC_65_ on P-gp ATPase activity was assessed in the recombinant human P-gp membranes. Change of luminescence signal in the presence of sodium vanadate (a P-gp ATPase inhibitor) represented the ATPase activity (control group). In the verapamil-treated group, the change of luminescence signal increased by approximately threefold greater than that of the vanadate-treated group, indicating an increase of the ATP hydrolysis from stimulation of P-gp ATPase activity (Fig. 6A). Our results also demonstrated that 600-HPTChC_65_ at the concentrations 0.32% (w/v) induced a significant change in luminescence signal, reflecting an increased ATPase activity (Fig. 6A). These findings suggested that 600-HPTChC_65_ might stimulate the P-gp ATPase activity.

### Effect of 600-HPTChC_65_ on the structure and expression of P-gp

**Figure 7:**
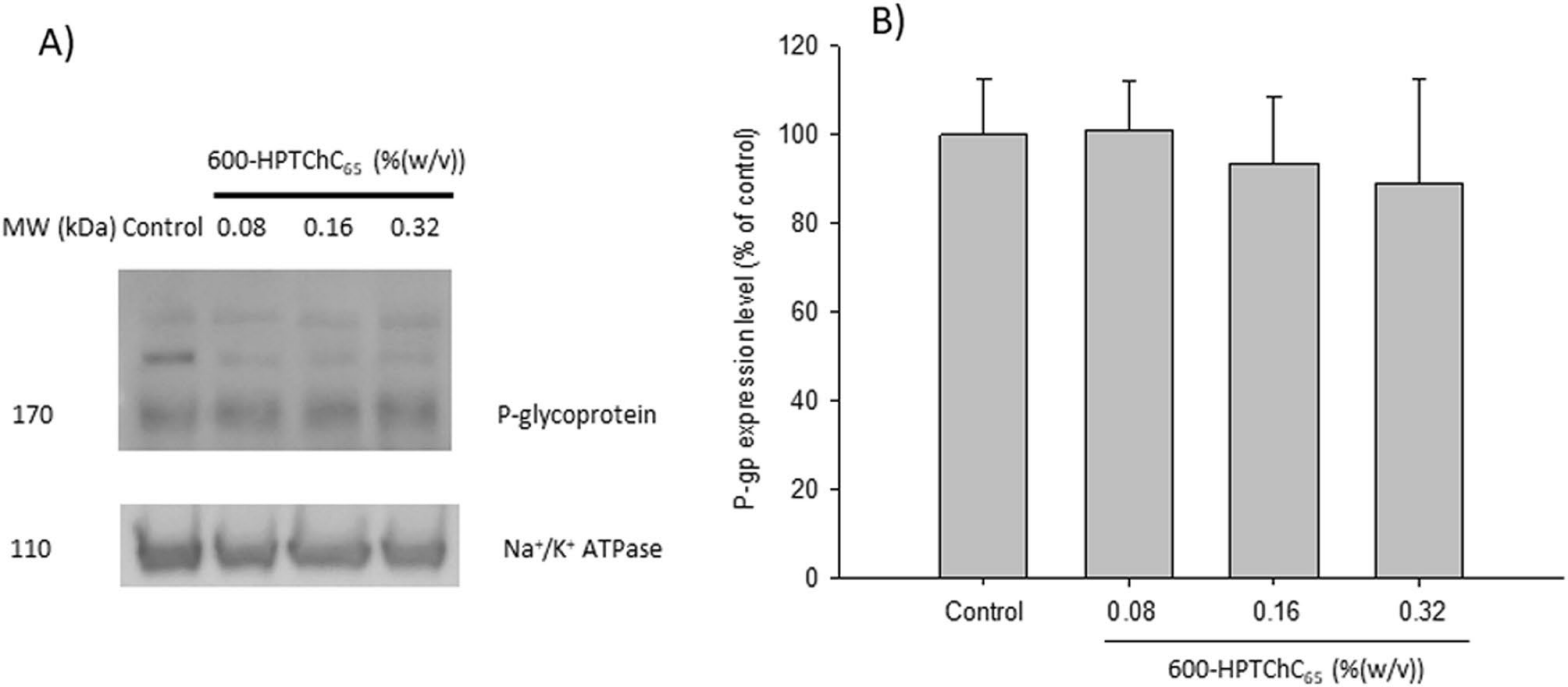
Effect of 600-HPTChC_65_ on P-gp expression after 1-day treatment in Caco-2 cells. (**A**) Western blot analysis and (**B**) densitometric analysis of P-gp expression. Na^+^/K^+^ ATPase was used as the internal standard. Each column represents the mean ± SEM (n = 3). The original images of immunoblot are presented in Supplementary Fig. S2.

The interaction between 600-HPTChC_65_ and P-gp expressed on the apical side of the Caco-2 monolayers was demonstrated by immunofluorescent imaging under a CLSM. As shown in Fig. 6B and C (white arrow), the green color of FITC-600-HPTChC_65_ overlapped with P-gp (red), suggesting the binding of FITC-600-HPTChC_65_ with P-gp protein. In addition, the direct binding of UIC2 monoclonal antibody to the extracellular epitope of P-gp in the UIC2 shift assay suggested the conformational change of P-gp protein in the 600-HPTChC_65_-treated group, as shown by a rightward shift of fluorescent signal of FITC after addition of FITC-conjugated UIC2 (Fig. 6D). These findings suggested that the 600-HPTChC_65_-treated cells displayed the higher number of UIC2-P-gp complexes, compared with the untreated group. However, 600-HPTChC_65_ even at the high concentration of 0.32% (w/v) had no effect on the expression of P-gp after 24-h treatment (Fig. 7).

## Discussion

Drug carriers have been utilized to enhance permeability of low bioavailability drugs across the intestinal barrier, leading to increasing the payload drug accessibility to the target sites and yielding a more effective treatment. Among various types of drug carriers, biopolymers such as chitosan and its quaternized derivatives have been widely used due to their outstanding biocompatibility, biodegradability and natural abundance. A series of HPTChC derivatives were synthesized in order to improve solubility and mucoadhesive properties^3,5^. The 200-HPTChC_53_ and 600-HPTChC_65_ derivatives displayed better biocompatibility with Caco-2 cells^3^. In this study, the superior ability of 600-HPTChC_65_ comparing to 200-HPTChC_53_ and unmodified chitosan on the enhanced Caco-2 monolayer permeability via the paracellular and transcellular pathways was further demonstrated.

In the paracellular pathway, 600-HPTChC_65_ significantly enhanced the tight junction opening to a greater degree than the lower MW 200-HPTChC_53_. These findings might be highly related to a greater mucoadhesive property of 600-HPTChC_65_, comparing with 200-HPTChC_53_. This explanation was supported by the higher Δ zeta potential of 600-HPTChC_65_ in mucin solution over the physiological pH range between 5.4 and 7.4. Hence, it was likely that 600-HPTChC_65_ generated a higher number of the positive surface charge than 200-HPTChC_53_. It was reported that increasing the cationic charge of the modified chitosan with cationic alkyl groups (i.e., trimethyl chitosan and dimethylethyl chitosan) markedly increased the paracellular permeability compared to the unmodified chitosan^19^. In addition, the MW of chitosan might contribute to the interaction between chitosan and biological barriers^20^. It was shown that the degree of interaction between chitosan and dipalmitoylphosphatidylcholine (DPPC) lipid bilayer increased in corresponding to increasing chitosan MW^21^. Comparing to 200-HPTChC_53_ and unmodified chitosan, 600-HPTChC_65_ could be a better absorptive enhancer due to its effect on tight junction opening as well as its better binding with the biological membrane.

Interestingly, 600-HPTChC_65_ suppressed the TEER values more rapidly than the unmodified chitosan 600 kDa, but the effect of these two compounds on the initial rate of FD-4 transport (within 50 min) was similar. Usually, TEER reflects the ionic conductance of the paracellular pathway across the epithelial monolayer, resulting from the permeation of ions and small molecules through size- and charge- selective pathways^22,23^. In contrast, the flux of non-electrolyte tracer (e.g., FD-4, inulin) across the barriers contributes to the paracellular water flow, the pore size and a leak pathway of the tight junction^24^. Hence, our findings suggested that 600-HPTChC_65_ interfered with an ionic flux faster than with the leak pathway, resulting in a more rapid rising ion permeability than that observed with unmodified chitosan.

The reversibility of tight junction opening and fast recovery rate of TEER values by 600-HPTChC_65_ may be one of the advantages of its uses. Loss of tight junction integrity has been associated with intestinal inflammation due to increasing the penetration of pro-inflammatory molecules, such as pathogens, toxins and antigens, into the mucosal tissue and blood circulation^25^. Cationic polymers that are being used as absorption enhancers, such as chitosan, poly-L-lysines and polyethyleneimine, can open the tight junction transiently, where tight junction integrity is completely restored to full function ability upon their removal^26^. The dynamics of tight junction opening and closing could be explained by the translocation of tight junction proteins, ZO-1 and occludin. It was possible that the disruption of tight junction and reversibility of tight junction opening by 600-HPTChC_65_ was related to ZO-1 and occludin reorganization, since upon removal of 600-HPTChC_65_, partial recovery of tight junction proteins (ZO-1 and occludin) was observed.

Our results suggested that internalization of 600-HPTChC_65_ into the cells was very low, possibly due to its large molecular size^27^. Regarding this, we hypothesized that 600-HPTChC_65_ might cause tight junction opening via either activation of intracellular signaling cascades or direct alteration of the tight junction protein complex. Several signaling pathways, such as MLCK, PKC and tyrosine kinase, are known to be related to tight junction opening^11,28^. However, the presence of specific inhibitors of these signaling cascades (i.e., ML-7, RO-318220 and genistein) did not prevent the effect of 600-HPTChC_65_ on tight junction opening, suggesting that this proceeded independently of the intracellular MLCK, PKC and tyrosine kinase signaling pathways. It was likely that 600-HPTChC_65_ directly interacted with tight junction proteins in the intercellular space, leading to the disassembly of the tight junction complexes. Previous studies have demonstrated that certain large molecules, such as peptides, which are difficult to translocate across the cell membrane, can induce tight junction permeability through interaction with the extracellular domain of occludin^29^. In this study, 600-HPTChC_65_ elicited a good mucoadhesive property, which could enhance its interaction with proteins at a neutral and basic pH. Hence, 600-HPTChC_65_ might induce tight junction permeability, in part, via alteration of the physical properties of the tight junction complex, such as the conformational structure and cell polarity. Regarding this, the mechanisms underlying tight junction opening by 600-HPTChC_65_ should be studied further.

It should be noted that, at 0.005% (w/v), 600-HPTChC_65_ might elicit its maximal effect on tight junction permeability, and thus the effect was not increased at a higher concentration of 0.02% (w/v). Possibly, it might be related to the saturation binding of 600-HPTChC_65_ on the cellular membrane that occurred even at such low concentration. Certain chitosan derivatives (e.g., MW 170 with 65% of deacetylation) also exhibited their limitation in enhancing mannitol absorption upon increasing their concentrations^6^.

In the transcellular pathway, P-gp activity has a significant impact on the drug absorption, especially low drug permeability in the intestine. Various excipients displayed their different behaviors in P-gp inhibition and drug transport. Several excipients with a significant fluidizing effect on the lipid bilayers such as Tween 80, Cremophor EL and polyethylene glycol (PEG) increased the AP-to-BL permeability of P-gp substrates (rhodamine 123, paclitaxel and doxorubicin)^15,30^. In contrast, some rigidizing membrane surfactant such as vitamin E TPGS (α-tocopheryl polyethylene glycol 800 succinate) reduced the BL-to-AP permeability of rhodamine 123 without alteration of the AP-to-BL permeability^30^. Our results showed that 600-HPTChC_65_ was able to slightly reduce the efflux ratio of [^3^H]-digoxin, suggesting its interference on P-gp function. Apparently, our synthetic polymer displayed its interference on both absorptive (AP-to-BL) and secretive (BL-to-AP) permeabilities of [^3^H]-digoxin. It has been well established that efflux activity of P-gp requires ATP hydrolysis from P-gp ATPase. Several polymeric materials such as thiolated chitosan, P85, Tween 80, cremophor EL and TPGS 1000 inhibited P-gp function and decreased the P-gp ATPase activity^31^. In this study, 600-HPTChC_65_ at the concentration of 0.32% (w/v) was able to increase the P-gp ATPase activity in the recombinant human P-gp membrane fraction. Hence, its suppressive effect on P-gp function might not stem from its inhibition against P-gp ATPase activity. Furthermore, unlike some excipients (e.g., peceol and gelucire 44/14), 600-HPTChC_65_ had no effect on the expression level of P-gp. Besides in vitro study, the in vivo experiment also demonstrated that polymeric P-gp inhibitor and thiolated chitosan significantly increased the oral bioavailability of P-gp substrate in rats^32^.

According to our UIC2 shift assay, 600-HPTChC_65_ was able to increase UIC2 reactivity, indicating that its interaction with P-gp presenting on the surface of Caco-2 monolayers resulted in the conformational change of P-gp. This effect might contribute to its ability to interfere with P-gp activity. Upon binding to P-gp, either substrates (e.g., rhodamine 123) or modulators (e.g., verapamil) changed the conformation of extracellular domain of P-gp, and subsequently induced the UIC2 shift^33^. Nevertheless, P-gp ATPase inhibitors (e.g., vitamin E TPGS) were reported to reduce UIC2 reactivity^34^. Taken together, these findings supported that binding of 600-HPTChC_65_ with P-gp would not result in inhibition of ATPase activity. Since upregulation of P-gp can occur in some circumstances such as in cancer environment, intestinal inflammation from overactive immune system or exposure to P-gp inducers (e.g., probiotics, *St. John*’s wort extract), it may reduce the bioavailability or drug uptake of P-gp drug substrates^35–38^. Therefore, the interference of 600-HPTChC_65_ on P-gp function demonstrated in our study may impact on the absorption enhancement of drugs that the bioavailability is limited by this efflux transporter.

## Conclusion

High MW 600-HPTChC_65_ displayed good potential as an absorption enhancer. This synthetic derivative of chitosan effectively increased paracellular transport via tight junction opening with full recovery. In addition, it was able to reduce P-gp activity through alteration of protein conformation. These dual mechanisms of 600-HPTChC_65_ may contribute to oral drug delivery system as an excipient with absorption enhancement effect. Further in vivo investigation should be pursued to confirm the absorption enhancement effect and safety of this chitosan derivative.

## Materials and methods

### Chemicals and reagents

Hank’s balanced salt solution (HBSS), 2-(N-morpholino) ethanesulfonic acid hydrate (MES), N-(2-hydroxyethyl)piperazine-N’-(2-ethanesulfonic acid) (HEPES), 3-(4,5-dimethylthiazol-2-yl)-2,5-diphenyltetrazolium bromide) (MTT), fluorescein isothiocyanate-conjugated dextran 4 kDa (FD-4), verapamil, [^3^H]-digoxin, mucin from porcine stomach type III, ML-7 (an MLCK inhibitor), RO-318220 (a PKC inhibitor), genistein (a tyrosine kinase inhibitor), Na^+^/K^+^ ATPase and 4’,6-diamidino-2-phenylindole dihydrochloride (DAPI) were purchased from Sigma-Aldrich (St. Louis, MO, USA). The clear-sol I scintillation cocktail and protease inhibitor cocktail were from Nacalai Tesque (Kyoto, Japan). The P-gp-Glo™ assay and P-gp antibody were obtained from Promega (Madison, WI, USA) and Alexis Biochemicals (San Diego, CA, USA), respectively. Primary antibodies against zonula occludens-1 (ZO-1) and occludin were from Invitrogen (San Diego, CA, USA). Goat anti-mouse IgG H&L Alexa Fluor® 488 and 568 secondary antibodies, FITC-labeled anti-P-gp [UIC2] monoclonal antibody and horseradish peroxidase (HRP)-conjugated secondary antibody were obtained from Abcam (Cambridge, UK). All other chemical reagents were analytical grades.

### Preparation of N-(2-hydroxypropyl)-3-trimethylammonium chitosan chloride (HPTChC)

The HPTChCs were synthesized from chitosan with a weight-average MW of 200 or 600 kDa (Seafresh Chitosan (Lab), Chumphon, Thailand) in the degree of quaternization with 3-chloro-2-hydroxypropyltrimethylammonium (CTMAC) at 53% (200-HPTChC_53_) and at 65% (600-HPTChC_65_). The degree of deacetylation of 200-HPTChC_53_ and 600-HPTChC_65_ was 41% and 29%, respectively. The chemical characterizations of these two compounds were performed as described previously^3^. Labeling of 600-HPTChC_65_ with FITC was performed as reported^39^.

### Cell cultures

Human colon adenocarcinoma Caco-2 cell line was obtained from the American Type Culture Collection (Manassas, VA, USA). The cells were maintained in complete medium (CM; Dulbeco's modified Eagle's medium supplemented with 10% (v/v) fetal bovine serum, 100 units/mL penicillin, 100 µg/mL streptomycin, 1% (v/v) non-essential amino acids and 2 mM L-glutamine) at 37 °C in a humidified atmosphere of 5% CO_2_. Cells were grown at a density of 6 × 10^4^ and 1.5 × 10^5^ cells/cm^2^ on a transwell insert (0.4 µm pore size; Corning Life Sciences, Tewksbury, MA, USA) for 21 days and 14 days in paracellular and transcellular experiments, respectively. At the start of each experiment, the integrity of the Caco-2 monolayer was assessed by measurement of the transepithelial electrical resistance (TEER), using a chopstick-like electrode connected to a Millicell-ERS® (Millipore, Bedford, MA, USA). Cell monolayers with a TEER value of greater than 300 Ω cm^2^ were used in each experiment.

### Determination of paracellular permeability

#### TEER measurements

The 21 day-aged Caco-2 monolayers were incubated with transport buffers, i.e., 10 mM MES-HBSS, pH 6.5 on the apical side (the donor compartment) and 25 mM HEPES-HBSS, pH 7.4 on the basolateral side (the receiver compartment) for 30 min at 37 °C. Subsequently, the test chitosan in 1% (w/v) acetic acid was added to the apical side and incubated at 37 °C on an orbital shaker for 4 h. To investigate the tight junction signaling pathway, the monolayers were incubated with either ML-7, RO-318220 or genistein for 30 min at 37 °C prior to addition of the test chitosan. The TEER values were monitored at the indicated time points during the treatment period.

For the measurement of tight junction recovery, each monolayer was incubated with the test chitosan for 1 h prior to replacement with CM for another 24 h in a CO_2_ incubator at 37 °C. The TEER value was measured at the indicated time points during the 24-h chitosan withdrawal period.

#### FD-4 transport

The 21-day aged Caco-2 monolayers were treated with 10 mM MES-HBSS containing the test chitosan and FD-4 (120 µg/mL) at the apical side at 37 °C for 4 h. At various time intervals, the basolateral buffer was taken to measure the fluorescence intensity of FD-4 at 485/535 nm (excitation/emission), using a microplate reader.

### Immunofluorescence microscopy of tight junction proteins, P-gp and FITC-labeled 600-HPTChC_65_ on the Caco-2 cell monolayer

Localization of tight junction proteins, P-gp and FITC-labeled 600-HPTChC_65_ on Caco-2 monolayers was determined by immunofluorescence microscopy^40^. After a 3-h treatment with either 600-HPTChC_65_ or its FITC-conjugated derivative, the monolayers were fixed with 1:1 acetone: methanol at 4 °C for 5 min, followed by 0.1% (v/v) Triton X-100 for 15 min, and 1% (w/v) bovine serum albumin for another 45 min. Then, the cells were incubated with anti-ZO-1 or anti-occludin antibodies (1:100) at 4 °C overnight, or anti-P-gp antibody (1:200) at room temperature for 1 h. Subsequently, the cells were incubated with the respective secondary antibodies (i.e., Alexa Fluor® 488 or Alexa Fluor® 568), and visualized under confocal laser scanning microscopy (CLSM; Fluoview FV10i; Olympus, Tokyo, Japan). The interaction of FITC-labeled 600-HPTChC_65_ with the cell monolayer was determined after staining with DAPI (1 µg/mL) for 10 min, and visualized under CLSM.

### Determination of the mucoadhesive property

The mucoadhesive property of chitosan and the chitosan derivatives was determined by measurement of the zeta potential of mucin at three different pH values (5.4, 6.5 and 7.4)^18^. In brief, the test chitosan at a final concentration of 0.02% (w/v) was added to 0.05% (w/v) mucin solution. The change in the zeta potential of mucin solution was measured at 25 °C, using a Zetasizer Nano ZS (Malvern Instruments, Malvern, UK).

### Transcellular transport study

Effects of 600-HPTChC_65_ on transcellular permeability were determined in the bidirectional transport study of [^3^H]-digoxin ^41,42^. Briefly, [^3^H]-digoxin (7.5 µCi/mL) and the test chitosan were added in either the apical (AP) or basolateral (BL) side of the 21-day aged monolayers at 37 °C. A known P-gp inhibitor verapamil was used as a positive control. After a 2-h incubation period, samples were taken from the opposite compartment [i.e., 50 µL (BL-to-AP) and 100 µL (AP-to-BL)], and then diluted in 2 mL of clear-sol I scintillation cocktail for measurement of radioactive activity with a liquid scintillation counter (LSC-6100, Aloka, Tokyo, Japan). The apparent permeability coefficient (P_app_) of [^3^H]-digoxin was calculated from the following equation.

$$Papp = \left(\frac{dQ}{dt}\right)\left(\frac{1}{ACo}\right)$$

where  *P*_*app*_ = the apparent permeability coefficient (cm/min), *dQ/dt* = the rate of appearance of [^3^H]-digoxin on the apical or basolateral side (µg/min), *A* = the surface area of the monolayers (cm^2^), *C*_0_ = the initial concentration of [^3^H]-digoxin in the donor compartment (µg/mL).

The efflux ratio (ER) was calculated using the equation:

$${\mathrm{ER}}={\mathrm{P}}_{\rm app({BL}-{to}-{AP})}/{\mathrm{P}}_{\rm app({AP}-{to}-{BL})}$$

where *P*_*app* (BL-to-AP)_ = the apparent permeability coefficient in the BL-to-AP direction, *P*_*app* (AP-to-BL)_ = the apparent permeability coefficient in the AP-to-BL direction.

### P-gp ATPase assay

Effect of 600-HPTChC_65_ on P-gp ATPase activity was determined in recombinant human P-gp membranes with the use of the P-gp-Glo™ assay (Promega, Madison, Wisconsin), according to the manufacturer’s instruction. Briefly, the test compounds (namely, 600-HPTChC_65_; 500 µM verapamil (a P-gp ATPase stimulator); 250 µM sodium vanadate (a P-gp ATPase inhibitor) were incubated with 25 µg of recombinant human P-gp membranes at 37 °C for 5 min, followed by addition of 5 mM Mg-ATP for 40 min. Then, the sample was mixed with an ATP detection reagent for 20 min. The luminescence signal (Relative Light Unit, RLU) was measured with a microplate reader in luminescence detection mode (FilterMax F5 Molecular devices, Sunnyvale, CA, USA). Difference of RLU between the treated and untreated samples indicated the amount of ATPase-mediated ATP consumption.

### UIC2 shift assay

Effect of 600-HPTChC_65_ on P-gp conformational transition change was evaluated by UIC2 shift assay^34,43^. The 21-day aged Caco-2 monolayers were harvested by trypsinization, and then resuspended in 3% FBS phosphate buffered saline. The cells were incubated with the test chitosan for 30 min at 37 °C, followed by FITC-labeled anti-P-gp [UIC2] monoclonal antibody (1 µg) for another 30 min. Then, UIC2 antibody was removed by centrifugation at 2300×*g*. The fluorescence intensity was measured by BD FACSAria™ II flow cytometer, equipped with FCS express 6 plus research edition. The result of each experiment was gated from 10,000 events. In this study, verapamil (100 µM) was used as a positive control.

### Western blot analysis

Protein expression of P-gp was determined with western blot analysis. The 14-day aged Caco-2 monolayers were treated with 600-HPTChC_65_ for 24 h, and then lysed with ice-cold 1% (v/v) protease inhibitor cocktail in HBSS. The membrane fraction was prepared by sequential centrifugation of the cell lysate at 828×*g* for 10 min and at 33,000×*g* for 1 h at 4 °C. The remaining cell pellet was collected and quantified the amount of protein with Bio-Rad protein assay kit II (Bio-rad Laboratories, Hercules, CA, USA). Protein samples (20 µg) were separated by 10% (w/v) sodium dodecyl sulfate–polyacrylamide gel electrophoresis (SDS-PAGE), and then transferred to a polyvinylidene difluoride (PVDF) membrane. The PVDF membrane was divided into 2 pieces at approximate MW of 130 kDa protein. After blocking with 5% (w/v) skim milk, the proteins were probed with anti P-gp antibody (1:1000) and anti Na^+^/K^+^ ATPase antibody (1:5000) (internal standard) at 4 °C overnight, followed by HRP-conjugated secondary antibody (1:5000) for 1 h at room temperature. The chemiluminescent signals were developed using ECL western blotting detection reagents, captured with an ImageQuant LAS 4000 (GE Healthcare Biosciences, Japan), and quantified with ImageJ software (NIH, Bethesda, MD, USA).

### Data analysis

Data are reported as the mean ± standard error of the mean (SEM) derived from at least three independent experiments. Statistical analysis was performed by either independent *Student*’s t-test or one-way analysis of variance (ANOVA) with subsequent Dunnett’s post-hoc analysis, where appropriate. A *p* value of less than 0.05 indicated statistical significance.

## Supplementary Information

Supplementary Information 1.

Supplementary Information 2.

Supplementary Information 3.

## Data Availability

All data generated or analyzed during this study are included in this published article and its Supplementary Information file.
