# Supplementary material for: Quaternization of high molecular weight chitosan for increasing intestinal drug absorption using Caco-2 cells as an in vitro intestinal model
Source: Sci Rep. 2023 May 16;13:7904. doi: 10.1038/s41598-023-34888-0 (PMC10188607; doi:10.1038/s41598-023-34888-0)
Supplement: Supplementary file 1 — Supplementary Information 1. [file 41598_2023_34888_MOESM1_ESM.pdf]

SUPPLEMENTARY FIGURE (S1)

Quaternization of high molecular weight chitosan for increasing intestinal drug absorption using Caco-2 cells as an in vitro intestinal model.

Ratjika Wongwanakul[a], Sasitorn Aueviriyavit[b,]*, Tomomi Furihata[c], Pattarapond Gonil[b], Warayuth Sajomsang[b], Rawiwan Maniratanachote[d], Suree Jianmongkol[a,]*

[a]Department of Pharmacology and Physiology, Faculty of Pharmaceutical Sciences, Chulalongkorn University, Bangkok, Thailand

[b]National Nanotechnology Center, National Science and Technology Development Agency, Pathum Thani, Thailand

[c]Laboratory of Pharmacology and Toxicology, Graduate School of Pharmaceutical Sciences, Chiba University, Chiba, Japan

[d]Toxicology and Bio Evaluation Service Center, National Science and Technology Development Agency, Pathum Thani, Thailand

Current Address (R Wongwanakul): National Nanotechnology Center, National Science and Technology Development Agency, Pathum Thani, Thailand.

***Corresponding authors:**

1: Suree Jianmongkol, Ph.D. (ORCID NUMBER: 0000-0002-2919-2339)

Department of Pharmacology and Physiology, Faculty of Pharmaceutical Sciences, Chulalongkorn University, 254 Phayathai Road, Bangkok 10330, Thailand

Telephone: +662-218-8318   E-mail ID: suree.j@pharm.chula.ac.th

2: Sasitorn Aueviriyavit, Ph.D.

National Nanotechnology Center, National Science and Technology Development Agency, 111 Thailand Science Park, Pathum Thani 12120, Thailand.

Telephone: +662-564-7100 Ext. 6566 E-mail ID: sasitorn@nanotec.or.th

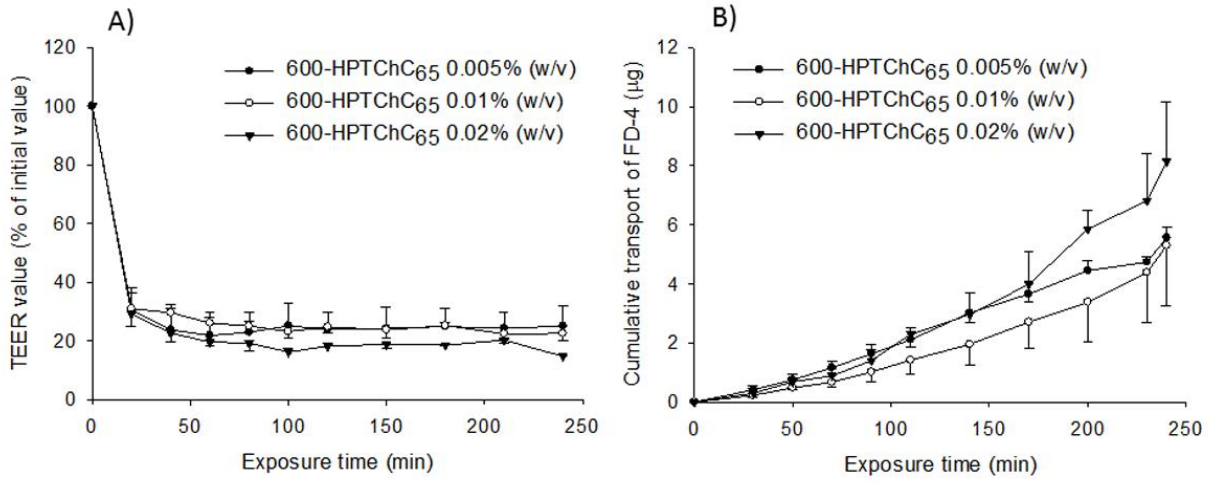

Supplementary Fig. S1. Paracellular permeability of Caco-2 monolayers after 4-h treatment with various concentrations [0.005–0.02% (w/v)] of 600-HPTChC$_{65}$. Tight junction integrity was assessed by (a) alteration of the TEER value (% of initial value) and (b) amount of FD-4 transport (µg). Data are expressed as the mean ± SEM (n= 3).
