# Supplementary material for: Quaternization of high molecular weight chitosan for increasing intestinal drug absorption using Caco-2 cells as an in vitro intestinal model
Source: Sci Rep. 2023 May 16;13:7904. doi: 10.1038/s41598-023-34888-0 (PMC10188607; doi:10.1038/s41598-023-34888-0)
Supplement: Supplementary file 2 — Supplementary Information 2. [file 41598_2023_34888_MOESM2_ESM.pdf]

SUPPLEMENTARY FIGURE (S2)

Quaternization of high molecular weight chitosan for increasing intestinal drug absorption using Caco-2 cells as an in vitro intestinal model.

Ratjika Wongwanakul[a], Sasitorn Aueviriyavit[b,*], Tomomi Furihata[c], Pattarapond Gonil[b], Warayuth Sajomsang[b], Rawiwan Maniratanachote[d], Suree Jianmongkol[a,*]

[a]Department of Pharmacology and Physiology, Faculty of Pharmaceutical Sciences, Chulalongkorn University, Bangkok, Thailand

[b]National Nanotechnology Center, National Science and Technology Development Agency, Pathum Thani, Thailand

[c]Laboratory of Pharmacology and Toxicology, Graduate School of Pharmaceutical Sciences, Chiba University, Chiba, Japan

[d]Toxicology and Bio Evaluation Service Center, National Science and Technology Development Agency, Pathum Thani, Thailand

Current Address (R Wongwanakul): National Nanotechnology Center, National Science and Technology Development Agency, Pathum Thani, Thailand.

***Corresponding authors:**

1: Suree Jianmongkol, Ph.D. (ORCID NUMBER: 0000-0002-2919-2339)

Department of Pharmacology and Physiology, Faculty of Pharmaceutical Sciences, Chulalongkorn University, 254 Phayathai Road, Bangkok 10330, Thailand

Telephone: +662-218-8318 E-mail ID: suree.j@pharm.chula.ac.th

2: Sasitorn Aueviriyavit, Ph.D.

National Nanotechnology Center, National Science and Technology Development Agency,

111 Thailand Science Park, Pathum Thani 12120, Thailand.

Telephone: +662-564-7100 Ext. 6566 E-mail ID: sasitorn@nanotec.or.th

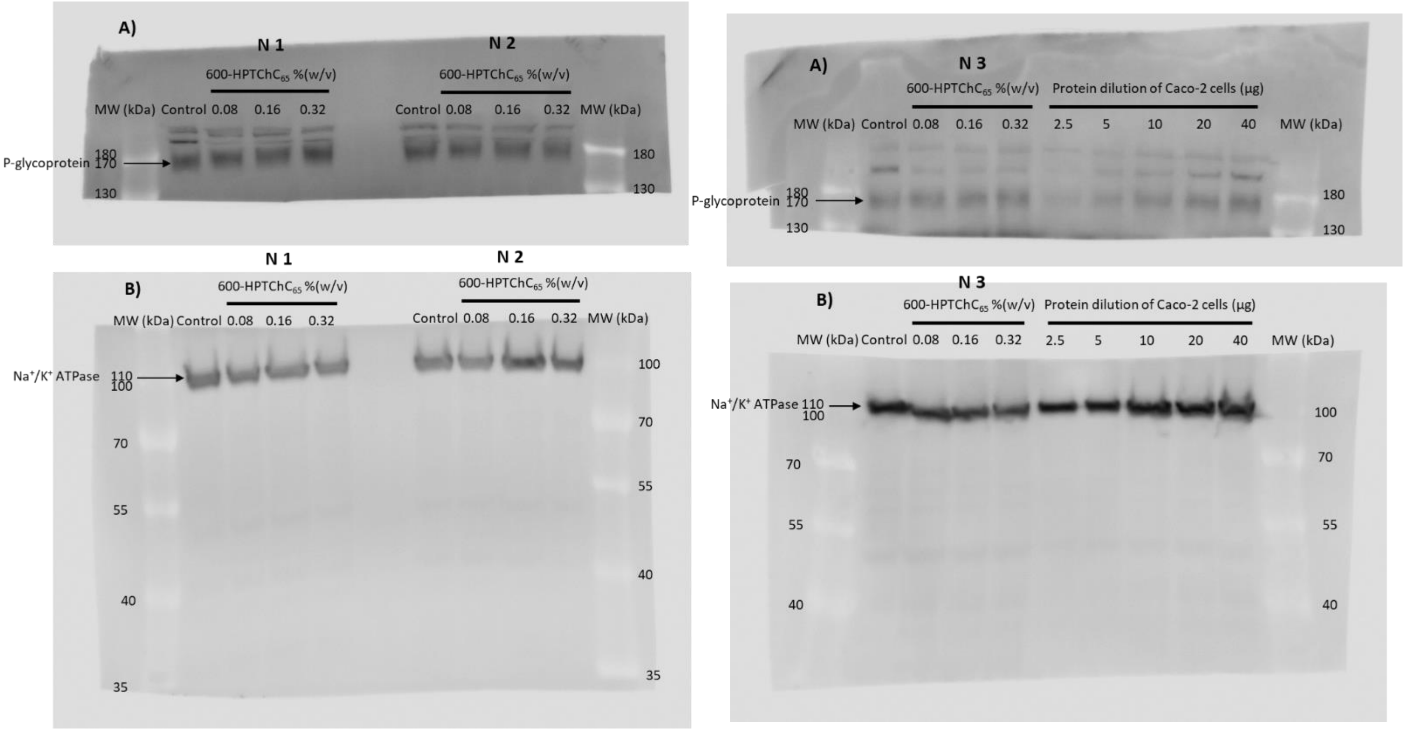

Supplementary Fig. S2. Immunoblots of protein samples (20 μg) from Caco-2 cells after 24-h exposure to 600-HPTChC$_{65}$ [0.08-0.32 % (w/v)] (n=3). After electroblotting, the PVDF membrane was divided into 2 pieces at approximate MW of 130 kDa protein. The upper piece **(A)** was probed with anti-P-gp, whereas the lower piece (**B)** was probed with anti-Na^+/K^+ ATPase (internal standard). The expression of P-gp and Na^+/K^+ ATPase in the untreated Caco-2 cells (control) at various amount of protein sample (2.5-40 μg) was also shown in the blot.
