# Supplementary material for: Quaternization of high molecular weight chitosan for increasing intestinal drug absorption using Caco-2 cells as an in vitro intestinal model
Source: Sci Rep. 2023 May 16;13:7904. doi: 10.1038/s41598-023-34888-0 (PMC10188607; doi:10.1038/s41598-023-34888-0)
Supplement: Supplementary file 3 — Supplementary Information 3. [file 41598_2023_34888_MOESM3_ESM.pdf]

SUPPLEMENTARY TABLE

Quaternization of high molecular weight chitosan for increasing intestinal drug absorption using Caco-2 cells as an in vitro intestinal model.

Ratjika Wongwanakul[a], Sasitorn Aueviriyavit[b,*], Tomomi Furihata[c], Pattarapond Gonil[b], Warayuth Sajomsang[b], Rawiwan Maniratanachote[d], Suree Jianmongkol[a,*]

[a]Department of Pharmacology and Physiology, Faculty of Pharmaceutical Sciences, Chulalongkorn University, Bangkok, Thailand

[b]National Nanotechnology Center, National Science and Technology Development Agency, Pathum Thani, Thailand

[c]Laboratory of Pharmacology and Toxicology, Graduate School of Pharmaceutical Sciences, Chiba University, Chiba, Japan

[d]Toxicology and Bio Evaluation Service Center, National Science and Technology Development Agency, Pathum Thani, Thailand

Current Address (R Wongwanakul): National Nanotechnology Center, National Science and Technology Development Agency, Pathum Thani, Thailand.

***Corresponding authors:**

1: Suree Jianmongkol, Ph.D. (ORCID NUMBER: 0000-0002-2919-2339)

Department of Pharmacology and Physiology, Faculty of Pharmaceutical Sciences, Chulalongkorn University, 254 Phayathai Road, Bangkok 10330, Thailand

Telephone: +662-218-8318 E-mail ID: suree.j@pharm.chula.ac.th

2: Sasitorn Aueviriyavit, Ph.D.

National Nanotechnology Center, National Science and Technology Development Agency, 111 Thailand Science Park, Pathum Thani 12120, Thailand.

Telephone: +662-564-7100 Ext. 6566 E-mail ID: sasitorn@nanotec.or.th

Supplementary Table S1. The zeta potential of submicron-sized mucin particles (SS-mucin) in the presence of 0.02% (w/v) chitosan 600 kDa or chitosan derivatives at different pH.

pH	Sample	ζ potential (mV)	Δ ζ potential (mV)
5.4	SS-mucin + 1% acetic acid	-3.99 ± 0.01	
	SS-mucin + 1% acetic acid + chitosan 600 kDa	+4.10 ± 0.63	+8.09 ± 0.63[a, b, c]
	SS-mucin	-4.34 ± 0.15	
	SS-mucin + 200-$HPTChC_{53}$	-0.92 ± 0.32	+3.43 ± 0.48[c, d]
	SS-mucin + 600-$HPTChC_{65}$	+7.18 ± 0.81	+11.52 ± 0.85[d]
6.5	SS-mucin + 1% acetic acid	-4.60 ± 0.08	
	SS-mucin + 1% acetic acid + chitosan 600 kDa	-2.68 ± 0.12	+1.92 ± 0.05[a, e]
	SS-mucin	-5.21 ± 0.07	
	SS-mucin + 200-$HPTChC_{53}$	-1.88 ± 0.03	+3.32 ± 0.12[e]
	SS-mucin + 600-$HPTChC_{65}$	+2.11 ± 1.55	+7.33 ± 1.49
7.4	SS-mucin + 1% acetic acid	-5.11 ± 0.10	
	SS-mucin + 1% acetic acid + chitosan 600 kDa	-3.55 ± 0.18	+1.56 ± 0.28[b, f, g]
	SS-mucin	-5.42 ± 0.22	
	SS-mucin + 200-$HPTChC_{53}$	-1.88 ± 0.33	+3.45 ± 0.15[f]
	SS-mucin + 600-$HPTChC_{65}$	+3.25 ± 1.55	+8.67 ± 1.43[g]

Data are expressed as the mean ± SEM (n =3). Means within a column followed by a different letter are significantly different ($p < 0.05$) (one-way ANOVA with post-hoc Dunnett's test).
